# *Pseudomonas aeruginosa* Exopolyphosphatase Is Also a Polyphosphate: ADP Phosphotransferase

**DOI:** 10.1155/2015/404607

**Published:** 2015-10-21

**Authors:** Paola R. Beassoni, Lucas A. Gallarato, Cristhian Boetsch, Mónica N. Garrido, Angela T. Lisa

**Affiliations:** Departamento de Biología Molecular, FCEFQyN, Universidad Nacional de Río Cuarto, Ruta 36 Km 601, Río Cuarto, 5800 Córdoba, Argentina

## Abstract

*Pseudomonas aeruginosa* exopolyphosphatase (*pa*Ppx; EC 3.6.1.11) catalyzes the hydrolysis of polyphosphates (polyP), producing polyP_n−1_ plus inorganic phosphate (P_i_). In a recent work we have shown that *pa*Ppx is involved in the pathogenesis of *P. aeruginosa*. The present study was aimed at performing the biochemical characterization of this enzyme. We found some properties that were already described for *E. coli* Ppx (*ec*Ppx) but we also discovered new and original characteristics of *pa*Ppx: (i) the peptide that connects subdomains II and III is essential for enzyme activity; (ii) NH_4_^+^ is an activator of the enzyme and may function at concentrations lower than those of K^+^; (iii) Zn^2+^ is also an activator of *pa*Ppx and may substitute Mg^2+^ in the catalytic site; and (iv) *pa*Ppx also has phosphotransferase activity, dependent on Mg^2+^ and capable of producing ATP regardless of the presence or absence of K^+^ or NH_4_^+^ ions. In addition, we detected that the active site responsible for the phosphatase activity is also responsible for the phosphotransferase activity. Through the combination of molecular modeling and docking techniques, we propose a model of the *pa*Ppx N-terminal domain in complex with a polyP chain of 7 residues long and a molecule of ADP to explain the phosphotransferase activity.

## 1. Introduction

polyP are linear polymers containing few to several hundred residues of orthophosphate linked by energy-rich phosphoanhydride bonds. The presence of this polymer has been detected in all kinds of living organisms, including higher organisms. Main enzymes involved in the synthesis of polyP are the polyphosphate kinases (Ppks; EC 2.7.4.1), which catalyze the formation of polyP from ATP (Ppk1) and GTP (Ppk2). Endo- and exopolyphosphatases are the most important enzymes involved in the utilization of polyP. In bacteria only exopolyphosphatases have been described [1].

The implication of Ppk and polyP in the virulence of* P. aeruginosa* has been clearly demonstrated, since a* ppk1* knockout mutant was defective not only in various forms of motility [2, 3] but also in biofilm development, quorum-sensing, synthesis of virulence factors such as elastase and rhamnolipid, virulence of the burned-mouse pathogenesis model [4], and general stress and stringent responses [5]. However, little is known about the relation between Ppx and its possible role in the pathogenesis of this bacterium [3, 6, 7]. We have recently confirmed that Ppx is relevant for pathogenesis in* P. aeruginosa* [8], due to the fact that the* ppx* null mutant was defective in the production of factors associated to both acute infection (e.g., motility-promoting factors, blue/green pigments production, and quorum-sensing C6–C12 homoserine lactones) and chronic infection (e.g., rhamnolipids and biofilm formation). Thus, there is enough evidence that both Ppk and Ppx as well as polyP balance contribute to the pathogenesis of* P. aeruginosa*.

Concentrations of polyP in* P. aeruginosa* are severalfold greater than in* E. coli* [9, 10].* P. aeruginosa* Ppx (*pa*Ppx) activity was described for the first time by Miyake and collaborators [11], and its gene was later cloned and expressed in* E. coli* [12]. The crystal structure and analysis of* E. coli* Ppx (*ec*Ppx) have been reported [13, 14] and the active site of the enzyme was suggested as a cleft where the polyP chain would bind. Several authors have determined that subdomains I and II of the N-terminal region of* ec*Ppx represent the catalytic portion, whereas the C-terminal region, formed by subdomains III and IV, was proposed to be involved in substrate binding [13–15].

The role of polyP as phosphate donor in phosphoryl transfer reactions has also been described [16]. Polyphosphate AMP phosphotransferase transfers a P_i_ from polyP to AMP producing ADP; afterwards, ATP is regenerated by the sequential action of the adenylate kinase. In addition, the glucose phosphotransferase catalyzes the phosphoryl transfer from polyP to glucose or glucosamine producing glucose 6-phosphate and glucosamine 6-phosphate, respectively. Finally, it is well known that Ppk is capable of catalyzing the inverse reaction to produce ATP from ADP + polyP (known as polyP:ADP phosphotransferase activity) (see [16] and references cited therein). To our knowledge, a similar activity of Ppx in prokaryotes has never been reported. Considering that other enzymes of the same family, such as the Ppx/GPPA of* Aquifex aeolicus* (*aa*Ppx; EC 3.6.1.40), act on a nucleotide (pppGpp) [17], we hypothesized that in the active site of* pa*Ppx there could be enough space to bind a nucleotide molecule such as ADP and that the reaction of ATP production using polyP as P_i_ donor could be possible. In the present work, we present results of the cloning and purification of* pa*Ppx, in full-length, N-terminal, and C-terminal domains variants and we describe the preference of the full-length enzyme for long polyP chains, pointing the C-terminal domain as responsible for this behavior. We show that* pa*Ppx is also a polyphosphate:ADP phosphotransferase and that the active site is the same as that one involved in the hydrolase activity. Finally, we present a structural model of full-length* pa*Ppx, in closed conformation, based on the atomic coordinates of* ec*Ppx [13] and a model of the N-terminal domain of* pa*Ppx in an open state based on atomic coordinates of* aa*Ppx [17]. We also propose a model of the* pa*Ppx N-terminal domain in complex with a polyP chain of 7 residues long and a molecule of ADP to explain the phosphotransferase activity through docking techniques.

## 2. Materials and Methods

### 2.1. Materials

Oligonucleotide primers were purchased from Integrated DNA Technologies (IDT, USA). ADP and sodium phosphate glass type 25, 45, 65, and 75 (polyP_25_, polyP_45_, polyP_65_, and polyP_75_) residues were purchased from Sigma (St. Louis, MO). The rest of the chemicals used were of pro-analysis quality.

### 2.2. Bacterial Strains and Growth Conditions

*P. aeruginosa* PAO1 and* E. coli* strains were grown in LB medium at 37°C. For recombinant* E. coli* the LB medium contained 150 *μ*g mL^−1^ ampicillin.* E. coli* XL10-Gold strain (Stratagene) was used for plasmid maintenance while* E. coli* BL21-CodonPlus strain (Stratagene) was used for protein expression.

### 2.3. DNA Methodology

DNA isolation, both genomic and plasmidic, was performed using commercial kits (Promega or Qiagen, resp.). Restriction enzymes and T4 ligase were used according to the manufacturer's instructions (Promega). DNA fragments were purified from agarose gels employing a QIAquick kit (Qiagen). To avoid introducing errors due to PCR or subcloning procedures, all resultant plasmids were partially sequenced by Macrogen, Inc. (Gangseo-gu, Seoul, South Korea).

### 2.4. Expression and Purification of* pa*Ppx Variants

The methods used were based on N-terminal fusion His-tagged proteins. The 1.5 Kb, 0.94 Kb, 0.91 Kb, and 0.56 Kb fragments encoding the 506 (*pa*Ppx_(1–506)_), 314 (N-*pa*Ppx_(1–314)_), 303 (N-*pa*Ppx_(1–303)_), and 192 (C-*pa*Ppx_(315–506)_) amino acids of Ppx were amplified from* P. aeruginosa* PAO1 wild-type chromosomal DNA through PCR with the oligonucleotides listed in Table 1. PCR-amplified DNA fragments were cloned into the pCR2.1-TOPO vector to generate pCR-*ppx*, pCR-N*ppx*, pCR-N303*ppx*, and pCR-C*ppx*, respectively. These plasmids were later transformed into* E. coli* XL10-Gold, followed by selection of ampicillin-resistant transformants. For gene expression, the different amplified* ppx* fragments were restricted by* Eco*RI-*Nde*I enzymes and subcloned into pET-15b (Novagen) as N-terminal fusions to a 6xHis-tag, generating pET-*ppx*, pET-N*ppx*, pET-N303*ppx*, and pET-C*ppx*, respectively. These plasmids were then transformed into* E. coli* BL21-CodonPlus. The resulting transformants were grown and induced as previously described by [18]. Affinity purification on Ni-agarose columns was used to perform protein purification, following the manufacturer's protocol (the QIA* expressionist*, Qiagen). Pure recombinant proteins were dialyzed against 10 mM Tris-HCl, pH 8.0, 150 mM NaCl, 150 mM imidazole, and 30% glycerol. Consecutive dialysis steps were performed to reduce the imidazole concentration to approximately 10 mM. The 6xHis-tag was subsequently removed using a thrombin cleavage capture kit (Novagen), and protein dialysis was repeated.

### 2.5. Enzyme Activities and Protein Assay

Ppx activity was measured after incubation at 37°C for 30 min in 200 *μ*L of 50 mM Tris-HCl buffer pH 8.0, 80 mM KCl, and 5 mM MgCl_2_. The substrates used were polyP_25_, polyP_45_, polyP_65_, and polyP_75_. The P_i_ released after incubation was measured by the Katewa and Katyare method [19] with modifications. Briefly, 100 *μ*L of the reaction mixture was added to 400 *μ*L of a solution with 2.5% (NH_4_)_6_Mo_7_O_24_·(H_2_O)_4_ in 3 N H_2_SO_4_ and 400 *μ*L of 2% ascorbic acid/2% hydrazine in 0.1 N H_2_SO_4_, and the solution was brought to a final volume of 1200 *μ*L with triple glass-distilled water. Quantification of free P_i_ was performed after 30 min of incubation at 37°C through measurement of the absorbance at 820 nm. One unit of exopolyphosphatase was defined as the amount of enzyme that releases 1 nmol of P_i_ per minute at 37°C.

The phosphotransferase activity was measured after incubation at 37°C for 30 min in 200 *μ*L of 50 mM Tris-HCl buffer pH 8.0, 200 *μ*M ADP, 8 *μ*M polyP_65_, and 5 mM of MgCl_2_ plus 80 mM KCl or 25 mM NH_4_Cl. ATP was determined using the luciferin-luciferase reaction (Kit A-6608, Molecular Probes). In sum, 50 *μ*L of the mixture described above was added to 450 *μ*L of 25 mM tricine pH 7.8, 5 mM MgSO_4_, 0.08 mM EDTA, 0.08 mM Na-azide, 10 mM DTT, 0.5 mM D-luciferin, and 10 *μ*g of firefly luciferase. The mix was incubated for 10 min and subsequently the emission spectra were measured between 500 and 650 nm, without excitement, with increments of 1 nm, an integration time of one second, and an emission slit of 10. The fluorescence measurements were performed in a Spex Fluoromax 3 spectrofluorometer (Jovyn-Ivon HORIBA). One unit of phosphotransferase was defined as the amount of enzyme that produces 1 nmol ATP per minute at 37°C. *K*_*M*_ and *V*_max_ values were estimated by nonlinear fitting of initial rate data according to the following equations: Hanes [*S*]/*v* = ([*S*]/*V*_max_) + (*K*_*M*_/*V*_max_); Michaelis-Menten (*v* = *V*_max_ [*S* or *M*])/*K*_*M*_ + [*S* or *M*]; and/or Hill ((*v*/*V*_0_ − *v*) = Log *K*_0.5_ + *n* log [metal ion]), where [*S*] and [*M*] correspond to substrate and metal ion concentrations, respectively. Protein concentration was determined by spectrophotometric measurement at 280 nm using the correspondent theoretical molar extinction coefficient calculated with the “ProtParam” tool [20] for physicochemical parameter prediction, which is available at the Expasy server (http://www.expasy.com/).

### 2.6. Molecular Modeling and Molecular Dynamics

The search of* pa*Ppx homologues through the use of the BLASTp algorithm resulted in the identification of an ortholog protein in* E. coli* (*ec*Ppx) with a 41% identity and 58% similarity. Two solved structures of this protein are available: PDB: 2FLO, 2.2 Å resolution [14] and PDB: 1U6Z, 1.90 Å resolution [13]. According to this, we used 1U6Z as template because the resolutions of the template had a large impact on the quality of the resulting model.

A homology model of* pa*Ppx in a closed conformation was constructed by comparative modeling using the ICM program [21]. For loop modeling, we performed a conformational sampling of these regions by means of the Sampling Loop module (Monte Carlo) implemented in ICM program.

The final model was validated using ProSA [22], ANOLEA [23], and PROCHECK [24]. Tautomeric states of histidine residues in the model were assigned according to the local environment using the Check Sidechains Plugin from VMD software [25]. The center of mass of the active site (residues Glu^126^, Asp^149^, Gly^151^, Ser^154^, and Glu^156^) was measured with VMD software, and in its place an atom of Mg^2+^, which is an essential cofactor, was added prior to the molecular dynamics.

The model of the N-*pa*Ppx_(1–314)_ in an open state was constructed using the atomic coordinates of PPX/GPPA phosphatase from* A. aeolicus* (*aa*Ppx) in complex with the alarmone ppGpp [26] (PDB: 2J4R). Considering not only the low percentage of identity between* pa*Ppx and* aa*Ppx [27] but also the homology in secondary structure, the model was constructed by threading using the “one-to-one threading” option of Phyre Server [27] (http://www.sbg.bio.ic.ac.uk/phyre2/). The model was obtained with 100% of confidence and 292 residues of a total of 314 were aligned. Tautomeric states of histidine residues in the model were assigned according to the local environment and Mg^2+^ ion was located in the active site similarly as we described for the full-length model. The obtained models were subjected to MD calculations to reach a minimum energy state. For this, the models were embedded in a 15 Å water box, and KCl 80 mM was used not only to mimic the optimal conditions for enzyme activity but also to neutralize the total charge of the system. The initial configuration of both systems was optimized using energy minimization followed by an equilibration through a molecular dynamics (MD) simulation in the NPT ensemble at 310°K for 1 ns, using a backbone restriction of 0.5 kcal/mol Å. NAMD program was used to perform all molecular dynamic simulations [28]. The electrostatic interactions were computed with no truncations using the particle mesh Ewald algorithm [29] under periodic boundary conditions.

The DYNDOM server was used to assess the opening degree model in open conformation with respect to the closed conformation [30]. By APBS software the potential electrostatic calculations were performed [31]. The charge and vdW radius assignment were determined with the software PDB2PQR [32] and the CHARMM force-field. pKa values were calculated using propKa [33].

### 2.7. Docking Assays

Docking studies were carried out as previously described [34], defining the N-*pa*Ppx_(1–314)_ model in open conformation as receptor. ICM [21] version 3.4 was used and the icmPocketFinder function was employed to detect possible binding sites with a tolerance of 4.6 by default. Tolerance is related to flexibility for sites prediction. The lower the tolerance value the higher the number of pockets predicted and vice versa. The value used was the one recommended by software developers.

Nucleotide ligand inputs were extracted from the PubChem database (http://pubchem.ncbi.nlm.nih.gov/) and the polyP ligand input was constructed by using ICM molecule editor. All ligand charges were assigned by ICM software. All of the structures were protonated and optimized using standard ICM protocols. The thoroughness parameter, which represents the length of the docking simulation, was set at 2.0, as recommended by software developers when metals are present in the docking binding site. For the docking of ADP, the gridbox size was 16.95 Å × 19.23 Å × 16.05 Å, and the center was located at the points (−5.602 × 7.844 ×  −4.172) Å. The binding site was determined by structural alignment with* aa*Ppx and the region at which ppGpp is located was used. Thus, the docking pocket was composed of the residues Asn^25^, His^28^, Gly^152^, Gly^224^, Asp^272^, and Arg^274^. For the docking of polyP_7_, the N-*pa*Ppx-ADP complex was used as receptor. The binding site was one of those detected by the icmPocketFinder tool and was consistent with the S-shaped canyon described by [14]. The binding site was constituted by the residues Tyr^94^; Asn^95^, Ser^122^, Gly^123^, Arg^124^, Glu^126^, Ile^130^, Asp^149^, Ile^150^, Gly^151^, Gly^152^, Gly^153^, Ser^154^, Glu^156^, Ser^170^, Gln^172^, Ser^223^: Gly^224^, Arg^227^: Ala^228^, Leu^231^, Gly^268^, Ile^269^, Lys^270^, Asp^272^, Arg^273^, Ile^276^, Glu^300^, Ala^302^, Leu^303^, Arg^304^, and Glu^305^. The gridbox size was 30.26 Å × 28.29 Å × 25.82 Å and the center was located at the points (−0.387 × 1.915 × −0.669) Å.

### 2.8. Sequence Analysis

To find orthologous proteins,* pa*Ppx sequence was used as query in PHMMER against UniProt rp55 database [35]. Sequences with an* E*-value ≤ 10^−14^ and a coverage percentage ≥ 77% were selected. The retrieved sequences (599) were aligned using Clustal Ω [36, 37]. Residues connecting N-terminal and C-terminal domains (residues 301 to 326) were selected to produce a Logo diagram conservation scheme, through the WebLogo server [38, 39] (http://weblogo.berkeley.edu/).

## 3. Results

### 3.1. Biochemical Characterization of* pa*Ppx

#### 3.1.1. Cloning and Overproduction of* pa*PPX Variants

The exopolyphosphatase gene of* P. aeruginosa* (*ppx*, PA5241) was cloned and overproduced as the full-length recombinant protein and also the two peptides representing the N-terminal and C-terminal domains. All recombinant proteins were purified and used to study some of the biochemical properties of the enzyme. The identification of N- and C-terminal domains was performed considering the crystallographic structure reported by [13, 14]. The full-length protein comprised 506 aminoacyl residues (*pa*Ppx_(1–506)_), while the N-terminal domain contained the first 314 aminoacyl residues and the remaining 192 aminoacyl residues corresponded to the C-terminal domain (N-*pa*Ppx_(1–314)_ and C-*pa*Ppx_(315–506)_, resp.). The theoretical MW for* pa*Ppx_(1–506)_, N-*pa*Ppx_(1–314)_, and C-*pa*Ppx_(315–506)_ were 56,419.33, 34,325.25, and 22,112.10 Da, respectively. Only* pa*Ppx_(1–506)_ and N-*pa*Ppx_(1–314)_ were enzymatically active while C-*pa*Ppx_(315–506)_ lacked enzymatic activity. The specific activities of* pa*Ppx_(1–506)_ and N-*pa*Ppx_(1–314)_ were 1.49 and 0.38 *μ*mol of P_i_ min^−1^ *μ*mol^−1^ protein, respectively. In order to determine the fragment responsible for the enzymatic activity of* pa*Ppx, we took into consideration the N-terminal construct obtained by [15], and we produced an N-terminal variant formed by the first 303 aminoacyl residues, named N-*pa*Ppx_(1–303)_, which were found to be inactive.

#### 3.1.2. Effect of the polyP Chain Length on* pa*Ppx Activity

**Figure 1:**
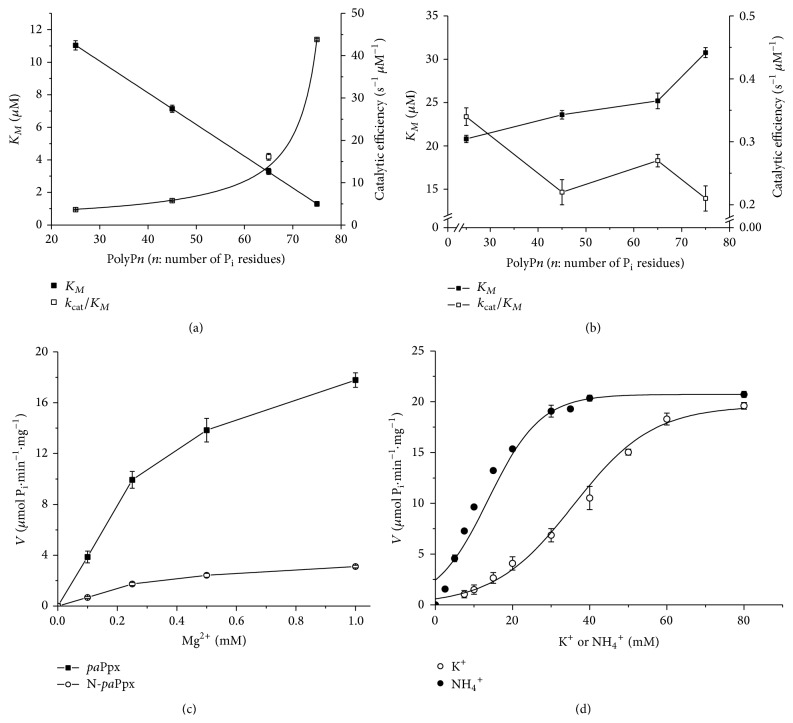
Kinetic characterization of* pa*Ppx_(1–506)_ and N-*pa*Ppx_(1–314)_. *K*_*M*(app)_ (■) and catalytic efficiency (*k*_cat_/*K*_*M*_) (□) of* pa*Ppx (a) and N-*pa*Ppx_(1–314)_ (b) for polyP of different chain length. Enzyme activity was measured in Tris-HCl buffer, pH 8.0, with Mg^2+^ 5 mM and 80 mM K^+^. (c) Saturation curves of* pa*Ppx_(1–506)_ (■) and N-*pa*Ppx_(1–314)_ (□) with Mg^2+^. Enzyme activity was measured in Tris-HCl buffer, pH 8.0, with polyP_65_8 *μ*M and 80 mM K^+^. (d) Saturation curves of* pa*Ppx with the monovalent ions K^+^ (○) and NH_4_^+^ (●). Enzyme activity was measured in Tris-HCl buffer, pH 8.0, with Mg^2+^ 5 mM and polyP_65_8 *μ*M.

The kinetic behavior evaluated and apparent catalytic constants obtained for* pa*Ppx_(1–506)_ and N-*pa*Ppx_(1–314)_ are displayed in Figure 1 and summarized in Table 2. With saturating Mg^2+^ and K^+^ concentrations,* pa*Ppx_(1–506)_ increased its affinity for polyP in the following order: polyP_25_ < polyP_45_ < polyP_65_ < polyP_75_. The decreasing *K*_*M*(app)_ values were accompanied by a sharp rise in the catalytic efficiencies, with this behavior being more noticeable with the increment in the length of the substrate chain (Figure 1(a)). This trend did not occur with N-*pa*Ppx since both *K*_*M*(app)_ and catalytic efficiency were similar independently of the length of the polyP chain (Figure 1(b), Table 2). Indeed, the analysis of the results obtained with polyP_75_ demonstrated that the full-length enzyme presented *K*_*M*(app)_ that was approximately 23-fold lower than the one of N-*pa*Ppx_(1–314)_ (1.30 ± 0.05 versus 30.67 ± 0.57 *μ*M, respectively). This behavior was partially reversed by an experiment of complementation, where a mixture of N-*pa*Ppx_(1–314)_/C-*pa*Ppx_(315–506)_, in a 1 : 10 ratio, produced an active enzyme with higher affinity for the substrate polyP_75_. The *K*_*M*(app)_ value measured in the mix was 11 ± 2 *μ*M, which represented an increase of ≈2.3-fold.

#### 3.1.3. Ion Dependence of Ppx Activity

*Divalent Ions Dependence*. We studied the behavior of the full-length* pa*Ppx_(1–506)_ and N-*pa*Ppx_(1–314)_ against different concentration of divalent ions such as Mg^2+^, Zn^2+^, Ca^2+^, and Mn^2+^ as effectors, in presence of a saturating concentration (8 *μ*M) for the substrate polyP_65_. The activation of both enzyme variants by Mg^2+^ was similar and showed no inhibition at high concentrations of this ion (Figure 1(c)). This result differed from the one obtained by [36, 37] for the* ec*Ppx activity that found a sharp decrease in the activity with Mg^2+^ concentrations of 1 mM and higher.

The values of *K*_0.5(app)Mg^2+^_ in* pa*Ppx_(1–506)_ and N-*pa*Ppx_(1–314)_ were 0.30 ± 0.02 mM and 0.28 ± 0.02 mM, respectively. Zn^2+^ was able to activate the enzyme only 20% compared to Mg^2+^, whereas the activation by Ca^2+^ and Mn^2+^ was negligible (3% and 2%, resp.). These data, added to the fact that the interaction between* pa*Ppx_(1–506)_ and Mg^2+^ occurs in the N-terminal domain, showed a clear preference of the enzyme for Mg^2+^ without inhibition by ion concentration up to 10 mM.

As expected, K^+^ was a nonessential activator of* pa*Ppx. This result is in good agreement with other studies performed on Ppxs [11, 15]. To assess the net effect of Mg^2+^, we performed a saturation curve of the divalent ion with the full-length enzyme, with and without K^+^. The presence of K^+^ did not affect the affinity of the enzyme for Mg^2+^; (*K*_0.5(app)Mg^2+^_ values were similar: 0.30 ± 0.02 mM (K^+^) and 0.33 ± 0.01 mM (no K^+^)).

*Monovalent Ions Dependence*. Considering the activation produced by K^+^ in the activity of* pa*Ppx_(1–506)_, we decided to test the effect of other monovalent ions. We observed that Li^+^ and Na^+^ presented no effects on enzyme activity while NH_4_^+^, K^+^, Rb^+^, and Cs^+^ were activators of* pa*Ppx_(1–506)_. Taking into account the physiological relevance of NH_4_^+^ and K^+^, saturation curves of* pa*Ppx with these ions were performed in the presence of 8 *μ*M of polyP_65_ and 5 mM of Mg^2+^. The curves obtained with NH_4_^+^ and K^+^ were sigmoid and reached their maximum activity at concentrations of 30 mM and 80 mM, respectively (Figure 1(d)). *K*_0.5(app)NH_4_^+^_ was 10 ± 0.4 mM and *K*_0.5(app)K^+^_ was 42 ± 0.5 mM for K^+^.

In view of the activation of* pa*Ppx_(1–506)_ by NH_4_^+^, we tested alkylammonium ions with different degrees of methylation as activators. We found that activation decreased as the number of methyl substituents increased. Considering the NH_4_^+^ activation as 100%, the percentages of activity with methylamine, dimethylamine, and trimethylamine were of 28, 9, and 5%, respectively. Tetramethylammonium was not an activator of* pa*Ppx_(1–506)_.

### 3.2. Phosphotransferase Activity

**Figure 2:**
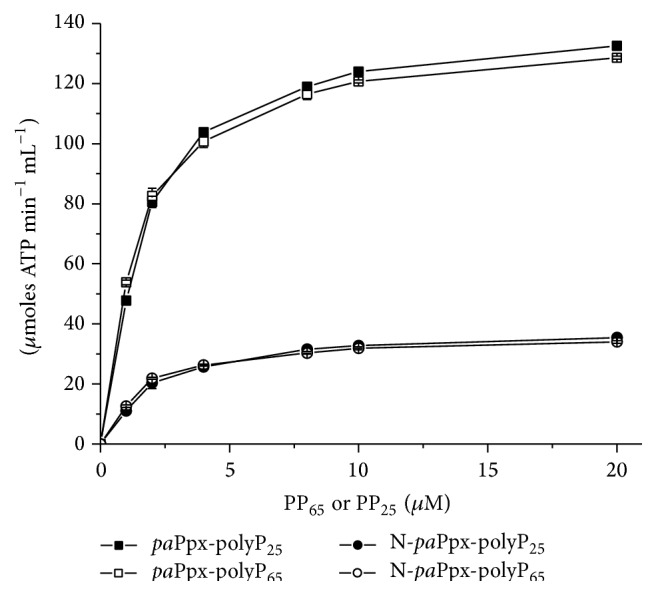
Phosphotransferase activity. Phosphotransferase activity of* pa*PPX_(1–506)_ (●, ○) and N-*pa*Ppx_(1–314)_ (■, □). Activity was measured with two substrates: polyP_25_ (●, ■) and polyP_65_ (○, □). Assayed conditions were Tris-HCl buffer, pH 8.0, 200 *μ*M ADP, 5 mM Mg^2+^, and 80 mM K^+^.

Since the Ppx/GPPA from* A. aeolicus* acts on a nucleotide (pppGpp) and the structure of this enzyme in presence of the product (ppGpp) (PDB: 2J4R) is available [17, 26], we hypothesized that in the active site of* pa*Ppx there could be enough space to bind a nucleotide molecule such as ADP; thus we decided to test the* pa*Ppx as a polyphosphate:ADP phosphotransferase. The production of ATP from polyP and ADP was measured in* pa*Ppx_(1–506)_ and N-*pa*Ppx_(1–314)_ with polyP lengths of 25 and 65 residues as substrates. Both variants presented phosphotransferase activity and, similarly to results obtained for the phosphatase activity, the N-*pa*Ppx_(1–314)_ had a lower catalytic efficiency (Figure 2). The catalytic parameters of the phosphotransferase activity compared to those of the phosphatase activity are listed in Table 2. In both variants, the phosphotransferase activity was independent of the polyP chain length since the *K*_*M*(app)_ and the *K*_cat_ were in the same order of magnitude for the polyP_25_ or the polyP_65_.

The phosphotransferase activity was dependent on Mg^2+^; however, the *K*_0.5(app)Mg^2+^_ value was approximately half that observed for phosphatase activity. The fact that the phosphotransferase activity was insensitive to the addition of K^+^ or NH_4_^+^ (Table 3) constituted an interesting finding. On the other hand, the affinity for ADP was independent of the length of the polyP chain used as phosphate donor. The *K*_*M*(app)_ value was in the order of 90 *μ*M for both substrates and variants tested (data not shown).

### 3.3. *In Silico* Studies

#### 3.3.1. Molecular Modeling of* pa*Ppx: Open and Closed Conformation

Initially, we modeled a full-length* pa*Ppx by comparative modeling, using the atomic coordinates of the crystal structure of* ec*Ppx [13] (PDB: 1U6Z). The template structure shared 41% of identity and 58% of similarity with* pa*Ppx_(1–506)_ and was reported in what the authors named “closed conformation.” In second place, we modeled the N-terminal domain of* pa*Ppx in the “open conformation” based on the atomic coordinates of* aa*Ppx [26] (PDB: 2J4R).

**Figure 3:**
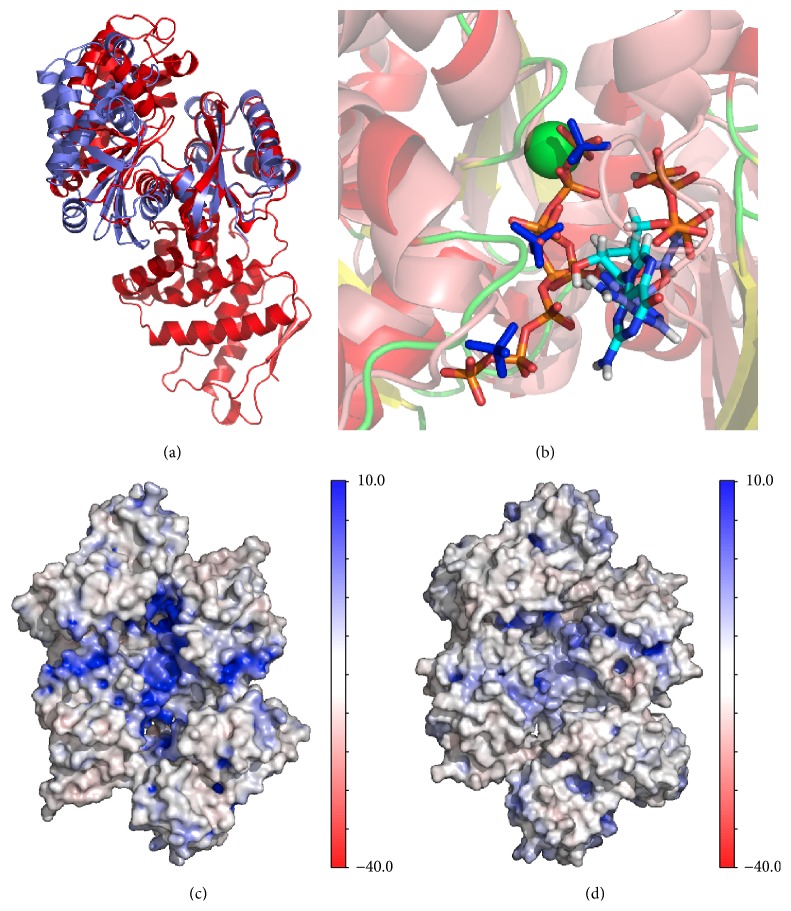
*In silico* studies. (a) Cartoon representation of the* pa*Ppx_(1–506)_ model in closed conformation superimposed with the N-*pa*Ppx_(1–314)_ model in open conformation. (b) Cartoon representation of the complex N-*pa*Ppx-ADP-polyP_7_ obtained by docking. A superimposition with sulfate ions (blue) of PDB: 1U6Z and ppGpp (Cyan) of PDB: 2J4R is shown. Mg^2+^ ion is represented as a green sphere. ((c) and (d)) Electrostatic potential calculated using APBS of* ec*Ppx (c) and* pa*Ppx (d) and mapped to the molecular surface, highlighting regions of positive potential.

Based on the homology in secondary structure and in spite of the low identity level between* pa*Ppx and* aa*Ppx (27%), the model was constructed by threading using the “one-to-one threading” option of Phyre Server [27]. The model was obtained with 100% of confidence and 292 residues of 314 were aligned. The resulting structures had an architecture that is characteristic within the actin-like ATPase domain superfamily, composed of two subdomains in the N-terminal domain and other two subdomains in the C-terminal (in the case of full-length* pa*Ppx). In the proteins of this family, movements of up to 30° were described to be related to the catalytic function of the enzymes. In* aa*Ppx a rotational movement of 22.5° between both domains around a single hinge region was described, indicating the access to the active site, located at the interface between domains. Kristensen and collaborators [26] described the access to the active site through a “butterfly-like” cleft opening.  Figure 3(a) shows the superimposition of N-terminal domains of the models of* pa*Ppx in the open and closed conformations. A rotation of 24° between subdomains I and II was detected by DYNDOM server between both structures [30], with G^123^:R^124^ and R^304^:E^305^ as bending residues.

#### 3.3.2. Active Site

By homology to what has been described in other exopolyphosphatases [13, 14, 17, 26], the active site of* pa*Ppx is formed by residues E^126^, D^149^, G^151^, S^154^, and E^156^. We confirmed the role of these amino acids in the active site of* pa*Ppx_(1–506)_ by performing nonconserved site-directed mutations, replacing individually each of these residues by alanine. The two activities, phosphatase and phosphotransferase, were measured and the release of both P_i_ and ATP production was severely affected (Table 4).

#### 3.3.3. Docking with polyP and ADP

After finding that* pa*Ppx can also act as a phosphotransferase, we were interested in proposing a three-dimensional model with polyP as P_i_ donor and ADP as the acceptor. Therefore, we performed docking assays that complemented our biochemical findings.

Sequential docking in the open conformation of N-*pa*Ppx_(1–314)_ was carried out. First, docking was performed with 7 residues long polyP (polyP_7_) chain in presence of Mg^2+^. Among the pockets detected by the IcmPocketFinder tool there was one that covered all the cleft, which was postulated to be opened or closed with N-terminal domain movements. Considering that this region was consistent with the location of the active site and the putative polyP binding sites described by [13, 14], we used this pocket for the docking assays. After the selection of the best conformer, the N-*pa*Ppx-polyP_7_ complex was used as the receptor molecule to perform docking with ADP. The possible conformations adopted by the nucleotide seemed to be led by the P_i_ moiety which is always located in the same position, and the guanosine group showed some degree of rotation resulting in slightly different positions inside the same binding site. The location of the terminal P_i_ nearby the Mg^2+^ atom was consistent with the proposed catalytic mechanism. It was also possible to see the rest of the polyP chain running along the cleft. The final complex is shown in Figure 3(b). The residues in the 4 Å radius of the ligands were Tre^95^, Asn^95^, Arg^98^, Ser^122^, Gly^123^, Arg^124^, Glu^126^, Ile^150^, Gly^151^, Gly^152^, Gly^153^, Ser^154^, Glu^156^, Ser^170^, Leu^171^, Gln^172^, Ser^223^, Gly^225^ Tre^225^, Arg^227^, Ala^228^, Leu ^231^, Lys^270^, and Arg^273^. polyP was stabilized by H-bonds with Gly^153^, Ser^154^, and Gly^224^ and saline bridges with Arg^227^, Lys^270^, and Arg^273^. Meanwhile, in the ADP, the ribose established an H-bond with Arg^304^ and *α* and *β* phosphates established an H-bond with Ser^24^ and Asn^25^. By comparison with the approach of Alvarado and collaborators [13], the model of N-*pa*Ppx_(1–314)_ in the open conformation with both ligands coexisting in the cleft was aligned with the N-terminal domain of* ec*Ppx, which had SO_4_^2−^ ions in the cleft. It is assumed that these ions describe a path for the polyP chain. The criterion for alignment was based on the active site residues, which remain in the same position regardless of the state of the conformation (opened or closed). This superimposition is shown in Figure 3(b). We also structurally aligned the complex obtained by docking with* aa*Ppx and it is noted that phosphates residues of 3′ are superimposable with the polyP residue while the 5′ phosphates residues are superimposable with 5′ phosphates of ADP (Figure 3(b)).

#### 3.3.4. Electrostatic Potential Calculations

A key aspect that remains to be characterized in the* pa*Ppx is the binding site of the polyP chain. Considering that the substrate of Ppx is a polyanion, it is possible that coulombic interactions govern the union between polyP and Ppx. Taking into account the relationship that may exist between the electrostatic potential of Ppx and the area where the polyP would bind [14], we performed the calculations of the electrostatic potential in* pa*Ppx_(1–506)_ model and* ec*Ppx. These results are shown in Figures 3(c) and 3(d). It is notorious that in* pa*Ppx the electropotential was much less positive than in* ec*Ppx, especially around the cleft.

## 4. Discussion

We have recently demonstrated the involvement of Ppx in the pathogenesis of* P. aeruginosa* [8]. This finding led us to investigate in greater detail the enzyme from a biochemical approach. Previous studies performed by several authors have established that the* ec*Ppx is composed of two independently folded domains: N- and C-terminal domains [13–15]. Based on this knowledge, we constructed several recombinant variants of* pa*Ppx:* pa*Ppx_(1–506)_, N-*pa*Ppx_(1–314)_, N-*pa*Ppx_(1–303)_, and C-*pa*Ppx_(315–506)_.

**Figure 4:**
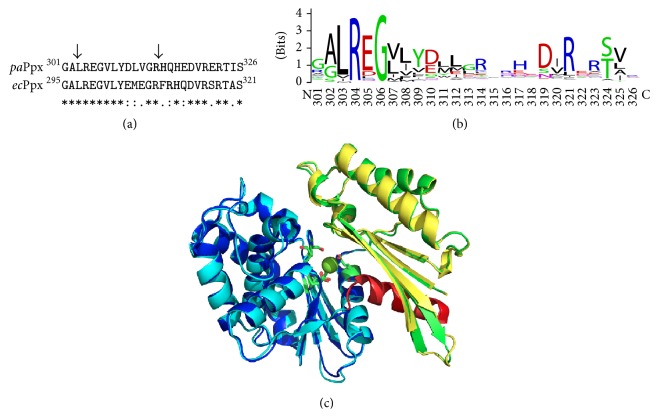
Sequence alignment of the connecting peptide between subdomains II and III. (a) Alignment of the end of N-*ec*Ppx_295–321_ and N-*pa*Ppx_301–326_. Sequences were aligned using ClustalX. The arrow indicates the cleavage site for both N-terminal variants used in this work N-*pa*Ppx_(1–314)_ and N-*pa*Ppx_(1–303)_. (b) Logo for* pa*Ppx peptide 301–326. Residues 303–315 constitute the last *α*-helix of N-terminal domain. Residues 316–320 constitute the loop connecting the N-terminal domain to the C-terminal domain. Residues 321–326 are forming the first *α*-helix of C-terminal domain. 599 sequences aligned with Clustal Ω were used to create the logo. (c) Cartoon representation of the closed conformation of N-*pa*Ppx in superimposition to N-*ec*Ppx (PDB: 1U6Z). Residues of active site are represented as sticks and Mg^2+^ ion is represented as a green sphere. Color references: N-*ec*Ppx: green: subdomain I, blue: subdomain II, and red: *α*-helix formed by residues 297–310. N-*pa*Ppx: yellow: subdomain I, cyan: subdomain II, and coral: *α*-helix formed by residues 303–315.

Our results indicate that the catalytic moiety of* pa*Ppx was localized in the N-terminal portion formed by the first 314 amino acid residues. Consistently with this, Alvarado and collaborators [13] also found that the N-terminal region of* ec*Ppx, formed by 320 amino acid residues, was responsible for the catalytic activity. However, Bolesch and Keasling [15] did not find* ec*Ppx activity in the N-terminal portion after limited proteolysis with* Staphylococcus aureus* V8 protease (Glu-C). A possible explanation for the discrepancy between these results may lie in the construction of the N-terminal variants. The peptide ^304^EMEGRFRHQDVRSRTAS^320^ located in the carboxyl end of the N-terminal variant constructed by Alvarado and collaborators [13] was absent in the N-terminal variant produced by Bolesch and Keasling [15]. This peptide constitutes a part of the connecting segment between domains II and III. When we compared the C-terminal end of the N-*ec*Ppx_295–321_ and N-*pa*Ppx_301–326_, we obtained a high degree of identity between these two enzymes (Figure 4(a)). The analysis of residue conservation through multiple sequence alignment showed that the residues forming the last *α*-helix of the N-terminal domain (297–304 in* ec*Ppx and 303–310 in* pa*Ppx) are conserved (Figure 4(b)). Structurally this helix is stepped between subdomains I and II of the N-terminal domain near the active site and constitutes a sort of separation between both subdomains (Figure 4(c)). We propose that this *α*-helix is involved in ligand interaction and/or folding of* pa*Ppx, since no enzymatic activity was detected in the variant N-*pa*Ppx_(1–303)_. Likely, the lack of this helix may prevent the proper folding of the domain or it may cause a disruption affecting the active site, resulting in loss of activity.

Although N-*pa*Ppx_(1–314)_ presented enzymatic activity, it had no preference for long polyP chains, and its *K*_*M*(app)_ and catalytic efficiency presented roughly the same values for all tested substrates. These results strongly suggest that the C-terminal domain is important for the recognition and/or interaction with long polyP chains. These findings are in concordance with those reported by [15] that stated that C-terminal domain of* ec*Ppx is involved in the recognition and processivity of long polyP chains.

The activity of* pa*Ppx was dependent on Mg^2+^, as it was demonstrated for other Ppxs [1]. Studies performed with the* ec*Ppx showed that the maximum activity was achieved in presence of Mg^2+^ 1 mM [35] while higher concentrations produced a sharp inhibition. It was reported for the Ppx of the archaea bacterium* Sulfolobus solfataricus* that Mn^2+^ is needed as an activator in concentrations ≈1 mM and that Mg^2+^ is needed to a lesser extent [38, 39]. Furthermore, for the mitochondrial Ppx of* Saccharomyces cerevisiae* [40] the requirements determined for divalent metals were similar: about 1 mM of Mg^2+^, Mn^2+^, and Co^2+^, including Zn^2+^ to a lesser extent. Results of these reports suggest similar catalytic mechanisms. Our results related to the activation of* pa*Ppx by divalent cations are in good agreement with the work of Dudev and Lim [41], who demonstrated that the Mg^2+^ binding sites are not often specific for Mg^2+^ and that Zn^2+^ may replace it in that location. We found that K^+^ and NH_4_^+^ are nonessential activators of* pa*Ppx. Our results considerably differ from those reported for the* ec*Ppx [36, 37], where more than twice the amount of K^+^ was necessary to achieve the maximal activity (175 mM versus 80 mM) and where ammonium sulfate was not considered as an activator of the* E. coli* enzyme. We believe that the lack of activation by ammonium sulfate may be due to the presence of sulfate ions which can bind to the enzyme and mimic the phosphate residues of the substrate, thus blocking the activity. The crystal structure reported by [13] also supports our inference, since numerous sulfate ions were bound to the enzyme, which led the authors to postulate that the presence of these ions represented the polyP chain path.

The whole data presented confirm that* pa*Ppx activity is dependent on Mg^2+^ and is stimulated by K^+^ in a similar manner to* ec*Ppx. Our present study also adds a new perspective to previous results, with the finding that NH_4_^+^ can act as a direct activator of* pa*Ppx at lower concentrations than K^+^. It was demonstrated that bacteria may accumulate transiently polyP during the stationary phase and especially under conditions of nitrogen and P_i_ limitation and during osmotic stress [9]. It is also known that K^+^ is the most prevalent cation in the cytoplasm and it becomes more concentrated as the osmolarity increases [42]. Therefore, in a hyperosmolar condition, the increase of K^+^ is useful to activate directly the* pa*Ppx. As the NH_4_^+^ concentration is limited or in the presence of a nonpreferential nitrogen source (amino acids, choline, etc.), the activation of* pa*Ppx is at transcriptional level since the expression of the* ppx* gene is under the control of the global regulator NtrC [8]. On the other hand, with a good availability of nitrogen in the environment, NH_4_^+^ directly stimulates the enzymatic activity and the bacteria obtain energy to start their growth.

Taking all these reports together, it was evident that polyP levels are involved in the global energetic state of the cell. Thus, we wondered if* pa*Ppx could have a second function, such as the synthesis of ATP by transferring P_i_ from polyP to a molecule of ADP. Confirming what we previously hypothesized, we found that* pa*Ppx is also a polyP:ADP phosphotransferase. As the phosphatase activity, the phosphotransferase activity is located in the N-terminal domain and depends on Mg^2+^; however, it is insensitive to the addition of K^+^ or NH_4_^+^, suggesting that the catalytic mechanism is somehow different, probably acting nonprocessively.

Additionally, we performed* in silico* assays to complement our experimental data. We modeled the full-length* pa*Ppx in a closed conformation and the N-*pa*Ppx in an open conformation.

In* E. coli*, the active site was proposed to lay in a cleft between subdomains I and II [13] and a glycine-rich phosphate-binding loop more likely stabilizes the transition state during catalysis [14].* pa*Ppx has full conservation of the proposed active site residues from* ec*Ppx that would be formed by Glu^156^, Asp^149^, Gly^151^, Ser^154^, and Glu^126^ and the glycine-rich loop consisting of residues Gly^141^-Ser^154^. The conserved Glu^126^ is proposed as the residue which activates a water molecule for the nucleophilic attack to the phosphodiester bond. The solved structures of* ec*Ppx lack the Mg^2+^ bound to the enzyme. The putative Mg^2+^ binding site was suggested by comparison with the binding of* aa*Ppx to a Ca^2+^ atom [17]. By analogy to the description of* ec*Ppx, the acidic residues Asp^149^ and Glu^156^ are predicted to contribute to the coordination sphere of Mg^2+^ in* pa*Ppx [14]. We confirmed the participation of Glu^126^, Asp^149^, Ser^154^, Gly^151^, and Glu^156^ residues in the active site by nonconservative site-directed mutagenesis in the full-length* pa*Ppx. The phosphotransferase activity of* pa*Ppx was also measured in the mutated variants of* pa*Ppx and no ATP production was detected. This result indicates that the active site of* pa*Ppx is the same for both activities: the phosphotransferase and the hydrolase.

It has been reported that* ec*Ppx and* aa*Ppx also presented pppGpp activity [17, 26, 43, 44], suggesting that the active site would have enough space to bind a nucleotide. Other authors [13, 14, 26] have debated whether there are two different active sites or a common one. Considering the work presented by Kristensen and collaborators [26] who have reported the structure of* aa*Ppx (in presence and absence of the alarmone ppGpp), it is clear that there is a single active site for hydrolysis of both polyP and pppGpp. As it was suggested by [14],* ec*Ppx must undergo a conformational change from the closed to the open state to allow the entry of the nucleotide pppGpp. A phosphotransferase activity was never described for exopolyphosphatases or PPX/GPPA phosphatases; this work is the first reporting ATP production in a Ppx. Our results suggest that in* pa*Ppx the same active site is responsible for this activity, and we think that the transition from the closed to the open conformation occurs also in* pa*Ppx to allow the entry of ADP. The pppGpp molecule in the active site is occupying the space that would occupy both polyP and ADP (Figure 3(b)). Considering that we propose that the active site for phosphotransferase and phosphatase activities is the same, we would expect that other Ppx/GPPA phosphatases also have the ability to synthesize ATP. A major aspect that remains to be characterized in the* pa*Ppx is the binding site of the polyP chain. This site is not well defined because so far there is no crystal structure of a Ppx bound to the substrate polyP. The polyP binding site has been suggested in* E. coli* by two different approaches. Alvarado and collaborators [13] have described a possible binding area based on several sulfate ions present in the crystal, assuming that sulfates may mimic phosphates. On the other hand, Rangarajan and collaborators [14] suggested the presence of a channel of highly positive electrostatic potential for polyP binding at the dimerization interface. Both approaches are consistent in the suggested areas for polyP binding to* ec*Ppx. These areas include the residues Arg^165^, Arg^166^, Arg^189^, and Lys^197^ corresponding to a monomer and His^378^, His^384^, Arg^383^, Arg^413^, Lys^414^, and Lys^488^ corresponding to the other monomer. Within these, it was found that Arg^166^, Lys^197^, His^378^, and Arg^413^ triads are linked to sulfate ions in a crystal. It is very striking that in* pa*Ppx only half of these residues are conserved. Thus, the channel formed after dimerization had a less positive electrostatic potential (Figure 3). Residues in this region of* pa*Ppx are Leu^171^, Gln^172^, Gln^195^, Glu^203^, His^383^, His^389^, Lys^388^, Arg^418^, Arg^419^, and Gln^491^. This leads us to believe that while there is a channel, the potential is much less positive and, therefore, there must be differences in the binding of both enzymes with polyP. One of the possible roles of K^+^ in the activity of* pa*Ppx may be the stabilization of the negative charges of polyP. This ion would also be involved in the constant attachment and detachment of the polymer during processive catalysis. If so, the interaction between the polyP and the enzyme in* ec*Ppx would be stronger than in* pa*Ppx and, thus, greater amounts of K^+^ would be required to detach the polyP in each catalysis cycle. We believe that this is one of the reasons why* ec*Ppx needs 175 mM of K^+^ for maximal activity whereas* pa*Ppx needs only 80 mM.

## 5. Concluding Remarks

In the present survey we show that, similarly to* ec*Ppx, the catalytic activity of* pa*Ppx is found in the N-terminal region formed by subdomains I and II. This N-terminal domain is unable to distinguish the long polyP chain of beyond 15 residues in length. As occurs with* ec*Ppx, the activity of* pa*Ppx depends on Mg^2+^ and is activated by K^+^. In addition, we found and described new and original properties of* pa*Ppx, including that the polypeptide connecting the *α*-helices from subdomains II and III is necessary for the catalytic activity and NH_4_^+^ is an activator of the enzyme and may work at lower concentrations than K^+^. Finally, we demonstrate that* pa*Ppx has also a phosphotransferase activity capable of producing ATP. Surprisingly, this activity is dependent on Mg^2+^ but is not activated by NH_4_^+^ or K^+^, suggesting that, in spite of the fact that the active site is the same, the catalytic mechanism is slightly different.

The regulation of the degradation of polyP is complex and dual, since it involves regulation at transcriptional and biochemical levels. Thus, bacteria have mechanisms which ensure that* pa*Ppx is active in various physiological situations such as (i) under nitrogen limitation, where* ppx* is activated by NtrC, mediated by a *σ* ^54^-dependent promoter [8]; (ii) under P_i_ deficiency mediated by PhoB by a *σ* ^70^-dependent promoter; (iii) in the presence of preferential nitrogen source (NH_4_^+^), where the transcription of* ppx* is inhibited but the existing enzyme can be directly activated; and (iv) under hyperosmolarity, where* pa*Ppx is rapidly activated by K^+^, the most prevalent cation in the cytoplasm. This enzyme with its two functions can release P_i_ for a direct synthesis of ATP or meet the nutritional needs where P_i_ is necessary either to generate more energy or to initiate metabolic processes.

Together, the foregoing data and observations point out that* pa*Ppx is an enzyme of remarkable relevance due to its implication in polyP metabolism and, consequently, in virulence and pathogenesis of* P. aeruginosa*. Despite the fact that several specific aspects related to the* pa*Ppx enzyme, such as the detection of the specific binding site of the polyP chain, remain to be characterized, our present work contributes to the understanding of activity of the enzyme and of some physiological aspects of* P. aeruginosa* as opportunistic pathogen.

## Figures and Tables

**(a):** 

Primer	Sequence 5′ → 3′
Upppx	GTCCATATGGACTTGCAAAGC
Dwnppx	TGCTTACCGCACGTTGAGGC
UpppxN	CATATGCACCAGCACGAGGAC
DwnppxC	CTAAGCGCCGACGAGGTCG
Up 304	GTCCATATGGACTTGCAAAGC
DWN 304	GACCAGTTAGTCGTAGAGAAC
E126A^*∗*^	CTCCGGCCGCGAG**GCA**GCCCGCCTGATC
D149A^*∗*^	CCGGCTGGTCAGC**GCC**ATCGGCGGCGGC
G151A^*∗*^	GGTCAGCGACATC**GCC**GGCGGCAGCACCG
S154A^*∗*^	CATCGGCGGCGGC**GCC**ACCGAGTTCATC
E156A^*∗*^	CGGCGGCAGCACC**GAG**TTCATCATCGGCC

**(b):** 

Strain or plasmid	Genotype and/or description	Reference or source
*P*. *aeruginosa* PAO1	Wild type	WUCG (2005)

*E*. *coli* XL10-Gold	Tet^r^Δ*(mcrA)183* *Δ(mcrCB-hsdSMR-mrr) 173 endA1 supE44 thi-1 recA1 gyrA96 relA1 lac*Hte [F′*proAB lacI* ^*q*^ *ZΔM15 Tn10* (Tet^r^) Amy Cam^r^]	Stratagene

*E*. *coli* BL21-CodonPlus	*E*. *coli* B F- dcm*ompThsdS*(rB- mB-) *gal*	Promega

pCR 2.1-TOPO	Amp^r^Km^r^; vector for TA cloning of PCR products	Invitrogen

pET-15b	Amp^r^, T7 promoter, multiple cloning sites, His-tag coding sequence	Qiagen

pCR-*ppx*	1.5 Kb *Eco*RI/*Nde*I fragment containing the *ppx* gene cloned into pCR 2.1-TOPO	This study

pCR-N*ppx*	0.94 Kb *Eco*RI/*Nde*I fragment containing the *ppx* gene cloned into pCR 2.1-TOPO	This study

pCR-N303*ppx*	0.94 Kb *Eco*RI/*Nde*I fragment containing the N-terminal domain of *ppx* gene cloned into pCR 2.1-TOPO	This study

pCR-C*ppx*	0.56 Kb *Eco*RI/*Nde*I fragment containing the *ppx* gene cloned into pCR 2.1-TOPO	This study

pET-*ppx*	1.5 Kb *Eco*RI/*Nde*I fragment containing the *ppx* gene cloned into pET-15b	This study

pET-N*ppx*	0.94 Kb *Eco*RI/*Nde*I fragment containing the N-terminal domain of *ppx* gene cloned into pET-15b	This study

pET-N303*ppx*	0.94 Kb *Eco*RI/*Nde*I fragment containing the N-terminal domain of *ppx* gene cloned into pET-15b	This study

pET-C*ppx*	0.56 Kb *Eco*RI/*Nde*I fragment containing the *ppx* gene cloned into pET-15b	This study

^*∗*^A pair of complementary primers was used to create each mutant. The sequence of the sense strand is shown. Changes in the sequences are shown in boldface type and underlined.

**Table 2:** Kinetic parameters of the full-length *pa*Ppx and the N-*pa*Ppx variant.

Parameters	Substrate	Hydrolase		Transferase	
		*pa*Ppx_(1–506)_	N-*pa*Ppx_(1–314)_	*pa*Ppx_(1–506)_	N-*pa*Ppx_(1–314)_
*K*_*M*(app)_ ± SD, *μ*M	polyP_75_	1.30 ± 0.05	30.67 ± 0.57	nd^2^	nd^2^
*k*_cat_^1^ ± SD, s^−1^	polyP_75_	57.02 ± 1.20	6.69 ± 0.26	nd^2^	nd^2^
*k*_cat_^1^/*K*_*M*(app)_ ± SD, s^−1^ *μ*M^−1^	polyP_75_	43.84 ± 0.19	0.22 ± 0.10	nd^2^	nd^2^

*K*_*M*(app)_ ± SD, *μ*M	polyP_65_	3.29 ± 0.19	25.17 ± 0.90	2.49 ± 0.20	3.11 ± 0.10
*k*_cat_^1^ ± SD, s^−1^	polyP_65_	53.03 ± 0.90	6.86 ± 0.17	3.93 ± 0.31	0.29 ± 0.04
*k*_cat_^1^/*K*_*M*(app)_ ± SD, s^−1^ *μ*M^−1^	polyP_65_	16.11 ± 0.80	0.27 ± 0.01	1.58 ± 0.13	0.09 ± 0.01

*K*_*M*(app)_ ± SD, *μ*M	polyP_45_	7.14 ± 0.12	23.60 ± 0.50	nd^2^	nd^2^
*k*_cat_^1^ ± SD, s^−1^	polyP_45_	41.23 ± 0.38	5.29 ± 0.07	nd^2^	nd^2^
*k*_cat_^1^/*K*_*M*(app)_ ± SD, s^−1^ *μ*M^−1^	polyP_45_	5.77 ± 0.15	0.22 ± 0.01	nd^2^	nd^2^

*K*_*M*(app)_ ± SD, *μ*M	polyP_25_	11.03 ± 0.29	20.83 ± 0.42	3.11 ± 0.1	3.36 ± 0.10
*k*_cat_^1^ ± SD, s^−1^	polyP_25_	40.20 ± 1.34	7.26 ± 0.16	4.28 ± 0.27	0.31 ± 0.02
*k*_cat_^1^/*K*_*M*(app)_ ± SD, s^−1^ *μ*M^−1^	polyP_25_	3.64 ± 0.12	0.35 ± 0.01	1.38 ± 0.12	0.09 ± 0.01

Values are means ± SD of at least three experiments performed independently.

^1^For *k*_cat_ determination, *pa*Ppx the full-length protein was considered as a dimer and N-*pa*Ppx was considered as a monomer.

^2^nd: not determined.

**Table 3:** Effect of mono- and divalent cations on phosphotransferase activity of the full-length *pa*Ppx and of the N-*pa*Ppx variant.

Mono- or divalent cations	*pa*Ppx_(1–506)_	N-*pa*Ppx_(1–314)_
	Phosphotransferase activity	Phosphotransferase activity
	(nmol ATP·min^−1^·mg^−1^)	(nmol ATP·min^−1^·mg^−1^)
Mg^2+^ (5 mM)	193.4 ± 6.6	39.3 ± 1.2
K^+^ (80 mM)	1.2 ± 0.1	0.9 ± 0.1
Mg^2+^/K^+^ (5 mM/80 mM)	187.6 ± 9.4	38.7 ± 2.5
NH_4_^+^ (25 mM)	2.1 ± 0.9	1.6 ± 0.4
Mg^2+^/NH_4_^+^ (5 mM/25 mM)	189 ± 10.1	42.9 ± 2.9

Values are means ± SD of at least three experiments performed independently.

**Table 4:** Hydrolase and transferase activity of mutated variants of *pa*Ppx.

Ppx variant	Hydrolase		Transferase	
	Specific activity^1^	%	Specific activity^2^	%
*pa*Ppx_(1–506)_	1.49 ± 4*E* ^−2^	100	10.92 ± 4*E* ^−1^	100
N-*pa*Ppx_(1–314)_	0.38 ± 6*E* ^−3^	25.5	1.34 ± 4*E* ^−2^	12.3
E126A^3^	0.016 ± 5*E* ^−5^	1.1	0.21 ± 9*E* ^−4^	1.9
D149A^3^	0.056 ± 7*E* ^−5^	3.8	0.31 ± 4*E* ^−4^	2.8
S154A^3^	0.079 ± 1*E* ^−4^	5.3	0.46 ± 1*E* ^−3^	4.2
G151A^3^	0.105 ± 4*E* ^−4^	7.1	0.69 ± 2*E* ^−3^	6.3
E156A^3^	0.034 ± 2*E* ^−4^	2.3	0.23 ± 9*E* ^−4^	2.1

Values are means ± SD of at least three experiments performed independently. Both activities were measured with 8 *μ*M polyP_65_, 5 mM of Mg^2+^, and 80 mM of K^+^.

^1^ *μ*mol of P_i_ min^−1^ *μ*mol protein^−1^.

^2^nmol of ATP min^−1^ *μ*mol protein^−1^.

^3^Mutated variants are all full-length *pa*Ppx_(1–506)_.

## Abbreviations

polyP: Polyphosphate
P_i_: Inorganic phosphate
pppGpp: Guanosine pentaphosphate
MD: Molecular dynamics
NPT: Isothermal-isobaric (NPT) ensemble
vdW: van der Waals.
